# In Vitro, In Vivo and In Silico Effectiveness of LASSBio-1386, an *N*-Acyl Hydrazone Derivative Phosphodiesterase-4 Inhibitor, Against *Leishmania amazonensis*

**DOI:** 10.3389/fphar.2020.590544

**Published:** 2020-12-16

**Authors:** Dahara Keyse Carvalho Silva, Jessicada Silva Teixeira, Diogo Rodrigo Magalhães Moreira, Tiago Fernandes da Silva, Eliezer Jesus de Lacerda Barreiro, Humberto Fonseca de Freitas, Samuel Silva da Rocha Pita, André Lacerda Braga Teles, Elisalva Teixeira Guimarães, Milena Botelho Pereira Soares

**Affiliations:** ^1^ Departamento de Ciências da Vida, Núcleo de Estudo e Pesquisa em Histopatologia, Universidade Estadual da Bahia (UNEB), Salvador, Brazil; ^2^ Laboratório de Engenharia Tecidual e Imunofarmacologia, Instituto Gonçalo Moniz, Fundação Oswaldo Cruz (FIOCRUZ), Salvador, Brazil; ^3^ Laboratório de Avaliação e Síntese de Substâncias Bioativas (LASSBio®), Universidade Federal do Rio de Janeiro (UFRJ), Rio de Janeiro, Brazil; ^4^ Laboratório de Bioinformática e Modelagem Molecular (LaBiMM), Faculdade de Farmácia, Universidade Federal da Bahia, Salvador, Brazil; ^5^ Departamento de Ciências da Vida, Laboratório de Modelagem Molecular Medicinal e Toxicológica, Universidade Estadual da Bahia (UNEB), Salvador, Brazil; ^6^ Instituto Senai de Inovação em Sistemas Avançados em Saúde, Senai/Cimatec, Salvador, Brazil

**Keywords:** *Leishmania amazonensis*, LASSBio-1386, treatment, N-acyl hydrazones, phosphodiesterase

## Abstract

Leishmaniasis are group of neglected diseases with worldwide distribution that affect about 12 million people. The current treatment is limited and may cause severe adverse effects, and thus, the search for new drugs more effective and less toxic is relevant. We have previously investigated the immunomodulatory effects of LASSBio-1386, an *N*-acylhydrazone derivative. Here we investigated the *in vitro* and *in vivo* activity of LASSBio-1386 against *L. amazonensis*. LASSBio-1386 inhibited the proliferation of promastigotes of *L. amazonensis* (EC_50_ = 2.4 ± 0.48 µM), while presenting low cytotoxicity to macrophages (CC_50_ = 74.1 ± 2.9 µM). *In vitro* incubation with LASSBio-1386 reduced the percentage of *Leishmania*-infected macrophages and the number of intracellular parasites (EC_50_ = 9.42 ± 0.64 µM). Also, *in vivo* treatment of BALB/c mice infected with *L. amazonensis* resulted in a decrease of lesion size, parasitic load and caused histopathological alterations, when compared to vehicle-treated control. Moreover, LASSBio-1386 caused ultrastructural changes, arrested cell cycle in G0/G1 phase and did not alter the membrane mitochondrial potential of *L. amazonensis*. Aiming to its possible molecular interactions, we performed docking and molecular dynamics studies on *Leishmania* phosphodiesterase B1 (PDB code: 2R8Q) and LASSBio-1386. The computational analyses suggest that LASSBio-1386 acts against *Leishmania* through the modulation of leishmanial PDE activity. In conclusion, our results indicate that LASSBio-1386 is a promising candidate for the development of new leishmaniasis treatment.

## Introduction

Leishmaniases are a complex of diseases caused by different species of protozoan parasites of the genus *Leishmania*, with worldwide distribution, affecting about 12 million people. Approximately two million individuals are infected annually, especially in tropical and subtropical countries, where about 350 million people reside in endemic areas (Burza et al., 2018; World Health Organization, 2020), accounting for approximately 30.000 deaths per year, and being considered the second leading cause of death due to parasite infection (Kevric et al., 2015; Pan American Health Organization/World Health Organization, 2017).

Pentavalent antimonials are the first-line drugs used in leishmaniasis treatment, and another chemotherapeutic agent, such as amphotericin B, paromomycin and pentamidine, can also be used as second-line. Although they are employed in several countries, these medicines are associated with several limitations, including painfulness and toxicity, long administration regimens and lack of compliance (Oliveira et al., 2011; Ponte-Sucre et al., 2017; Torres-Guerrero et al., 2017). Furthermore, careful supervision of health professionals is needed due to systemic adverse effects, such as myalgia and cutaneous rash, as well as to their high toxicity (Oliveira et al., 2011; Ponte-Sucre et al., 2017; World Health Organization, 2020). Although new drug formulations such as liposomal amphotericin B have been used to reduce the toxicity and showed great efficacy in leishmaniasis treatment, their high cost is still a limitation (Chakravarty and Sundar, 2019).

In this context, the development of new drugs more selective and effective against leishmania and less toxic to patients is of great relevance (Bhattacharya et al., 2020). The *N*-acylhydrazone (NAH) is considered a privileged chemical structure used as a template for derivatization of multi-target drugs (Duarte et al., 2007). Compounds with this subunit showed antiparasitic, antimicrobial, and immunomodulatory actions (Kümmerle et al., 2012; Alencar et al., 2014; Guimarães et al., 2018; Thota et al., 2018). A series of new furoxanyl *N*-acylhydrazones presented antimicrobial and antiparasitic activities against *Mycobacterium tuberculosis*, *Trypanosoma cruzi* and *Leishmania amazonensis* (Hernández et al., 2013). Furthermore, several studies have shown that NAH subunit is the pharmacophore group for inhibition of cysteine proteases, which are essential in several parasitic biochemical pathways (Li et al., 1996; Mckerrow et al., 1999; Ifa et al., 2000; Romeiro et al., 2009; Siqueira-Neto et al., 2018).

LASSBio-1386, a NAH derivative (*E*)-*N*'-(3,4-dimethoxybenzylidene)-4-methoxy-N-methylbenzohydrazide, presented a strong immunomodulatory action via NF-κB pathway inhibition and which could aid the inflammatory or immune-mediated diseases treatment (Guimarães et al., 2018). This compound promoted vascular hypertrophy in a pulmonary hypertension model (Alencar et al., 2014) and also showed potent inhibitory activity against human PDE-4 (*Hs*PDE4) and significantly decreased TNF-α release by lipopolysaccharide (LPS)-activated human blood mononuclear cells (Kümmerle et al., 2012).

It is known that *Hs*PDE inhibitors could also block *Lmj*PDEB1 and *Lmj*PDEB2 preventing *in vitro* proliferation of *L. major* promastigotes (Sebastián-Pérez et al., 2018). Previous studies *in vitro* and *in vivo* revealed that PDEs are essential for parasite survival and for infection maintenance (Card et al., 2004; Wang et al., 2007; Meira et al., 2017; Sebastián-Pérez et al., 2018). Additionally, our structural comparison analysis between *Hs*PDE4 and *Lmj*PDEB1 shown a high residue similarity mainly on each respective substrate recognition site (87%, Supplementary Figure 1S). Taken together, these data allowed us to suppose that LASSBio-1386 could act against *L. major* and *L. amazonensis* through PDE pathway modulation.

Thus, in this work, we studied the LASSBio-1386 activity against distinct forms of *L. amazonensis* through *in vitro* and *in vivo* assays as well as its molecular interaction with *Leishmania major* phosphodiesterase B1 (*Lmj*PDEB1) and we also discussed the implications for their selectivity.

## Materials and Methods

### Animals

Female, 6 to 12-week-old BALB/c mice (*Mus musculus*) were raised and maintained at the animal facilities of the Gonçalo Moniz Institute, Oswaldo Cruz Foundation, Salvador, Brazil. Animals were maintained in rooms with controlled temperature (22 ± 2 °C), humidity (55 ± 10%), continuous air renovation, 12 h light/12 h dark cycle and a balanced diet for rodents and water *ad libitum*. The protocol was approved by the Institutional Animal Care and Use Committee, Ethics Committee for Animal Experimentation of FIOCRUZ (CEUA/FIOCRUZ Permit Number: L-IGM-004/2019).

### Drugs

**FIGURE 1:**
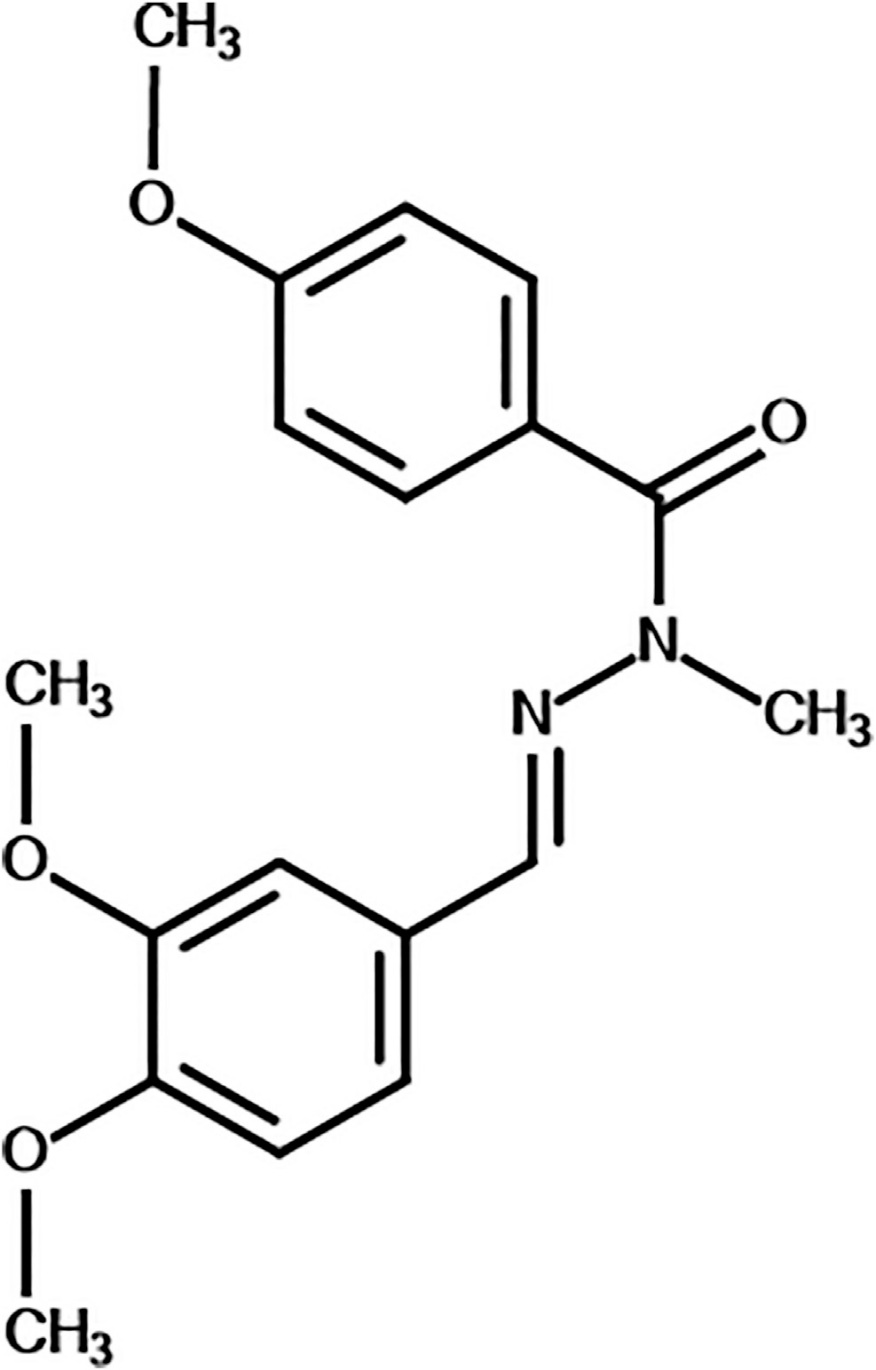
Chemical structure of LASSBio-1386.

LASSBio-1386 - (*E*)-*N*'-(3,4-dimethoxybenzylidene)-4-methoxy-N-methylbenzohydrazide (Figure 1) was synthesized by Laboratório de Avaliação e Síntese de Substâncias Bioativas at Federal University of Rio de Janeiro, Brazil, as previously described (Kümmerle et al., 2012). Gentian violet (Synth, São Paulo, SP, Brazil) was used as a positive control in the cytotoxicity assays. Amphotericin B (Life Technologies, GIBCO-BRL, Gaithersburg, MD), was used as a positive control in antileishmanial assays. All compounds were solubilized in dimethyl sulfoxide (DMSO) (PanReac, Barcelona, Spain) and for the use in the assays were diluted in cell culture medium. The final concentration of DMSO was <0.1% in all *in vitro* experiments and <5% in all *in vivo* experiments (Guimarães et al., 2018).

### Parasites

*L. amazonensis* promastigotes (MHOM/BR88/BA-125 Leila strain) were cultivated in *Liver Infusion Tryptose* (LIT) medium or *Schneider* (Sigma-Aldrich) medium supplemented with 10% fetal bovine serum (FBS; GIBCO), 50 μg ml^−1^ of gentamicin (Life, Carlsbad, CA), pH 7.2, at 26 °C. Daily counts of parasites culture was performed in a Neubauer chamber for five days. *In vitro* passages of culture were performed upon reaching stationary phase of growth and the infectivity of the parasites was maintained through passages in BALB/c mice.

### Cytotoxicity Evaluation

Cytotoxicity of LASSBio-1386 was determined from J774 immortalized macrophages. Macrophages (6.8 × 10^4^ cells/well) were incubated in Dulbecco's modified Eagle's medium (DMEM; GIBCO) supplemented with 10% FBS (GIBCO) and 50 μg ml^−1^ gentamicin (Life, Carlsbad, CA), for 24 h, at 37 °C and 5% CO_2_. LASSBio-1386 was added in sets of six concentrations diluted 1/3 (100–0.41 µM), in triplicates, and incubated for 24 h. Subsequently, 20 μL/well of AlamarBlue (Invitrogen, Carlsbad, CA) was added for 6 h at 37 °C and 5% CO_2_. Colorimetric readings were performed on the spectrophotometer at 570 and 600 nm. Gentian violet (Synth, São Paulo, SP, Brazil) was used as the reference cytotoxic drug (Meira et al., 2017; Da Silva et al., 2018). EC_50_ values were determined from data-points gathered from three independent experiments by using Graph Pad Prism version 5.01 (Graph Pad Software, San Diego, CA, United States).

### Viability Assay

Promastigotes of *L. amazonensis* (1 × 10^6^/well) were cultivated in Schneider's medium (Sigma-Aldrich) supplemented with 10% FBS (GIBCO) and 50 μg ml^−1^ gentamicin (Life, Carlsbad, CA). LASSBio-1386 was added in six concentrations of LASSBio-1386 diluted 1/3 (20–0.625 µM) for 72 h at 26 °C. Subsequently, 20 μL/well of AlamarBlue (Invitrogen, Carlsbad, CA) was added for 2 h. Colorimetric readings were performed on spectrophotometer at 570 and 600 nm. The percentage inhibition of compounds was determined based on the untreated control (Guedes et al., 2018). EC_50_ values were determined from data-points gathered from three independent experiments by using Graph Pad Prism version 5.01 (Graph Pad Software, San Diego, CA, United States).

### *In vitro* Macrophage Infection and Incubation With LASSBio-1386

The effect of LASSBio-1386 in intracellular parasites was assessed through macrophage infection. J774 macrophages (2 × 10^5^/well) were cultured and infected with promastigotes of *L. amazonensis* at stationary-phase on a ratio of 10:1, for 4 h. Infected cells were incubated with different concentrations of LASSBio-1386 (15, 30 and 45 µM) for 24 h. Amphotericin B was used as a positive control. The cells were fixed in methanol and stained by GIEMSA (Sigma-Aldrich). The percentage of infection and the number of intracellular parasites per macrophages were determined by counting 100 cells per slide under an optical microscope (Guimarães et al., 2006). EC_50_ for intracellular parasites was calculated using the number of intracellular parasites per macrophage. EC_50_ values were determined from data-points gathered from three independent experiments by using Graph Pad Prism version 5.01 (Graph Pad Software, San Diego, CA, United States).

### Cell Cycle Analysis

Quantification of the nuclear DNA content which reflects the phases of the cell cycle, was evaluated using propidium iodide (PI) as a fluorogenic agent. Promastigotes (1 × 10^7^/well) were incubated with LASSBio-1386 (15, 30 and 45 µM) for 48 h. Parasites were washed with saline, centrifuged for 10 min at 1700 g and diluted in the lysis solution containing PI (0.1% Triton X-100 and 2 μg ml^−1^ propidium iodide in PBS) in the absence of light at 37 °C. After 30 min, the samples were acquired on a LSRFortessa flow cytometer (Becton Dickinson Biosciences, San Jose, CA, United States) and analyzed by FlowJo software (Tree Star, Ashland, OR) (Verçoza et al., 2017).

### Mitochondrial Membrane Potential of Promastigotes of *L. amazonensis*

Promastigotes of *L. amazonensis* (10^6^ cells/mL) were incubated with LASSBio-1386 in different concentrations (EC_50_ or 2x EC_50_) for 72 h. After incubation, parasites were added in cytometry tubes with 10 µg ml^−1^ of rhodamine 123 (Sigma-Aldrich, St. Louis, United States) for 15 min. Methanol was used as a positive control. After incubation, data acquisition was performed using a LSRFortessa flow cytometer and the analysis was performed by FlowJo software (Aliança et al., 2017).

### Ultrastructural Analysis

Macrophages infected with *L. amazonensis* were incubated with LASSBio-1386 (30 and 45 µM) for 24 h at 37 °C and 5% CO_2_. After incubation, the cells were fixed for 1 h at room temperature with a solution of 2% formaldehyde and 2.5% glutaraldehyde (Electron Microscopy Sciences, Hatfield, PA, United States) in sodium cacodylate buffer (0.1 M, pH 7,2). Subsequently, the cells were washed with sodium cacodylate buffer (0.1 mM, pH 7.2) four times, and post-fixation were performed with a solution of 1% osmium tetroxide (Sigma Chemical Co., St. Louis, MO, United States). Cells were dehydrated in increasing concentrations of acetone (30, 50, 70, 90 and 100%) during 10 min for each step and embedded in Polybed resin (PolyScience family, Warrington, PA, United States). Ultrafine sections were prepared in Leica UC7 ultramicrotome, collected, contrasted with uranyl acetate and lead citrate and analyzed in a JEOL TEM-1320 transmission electron microscope (Rocha et al., 2013).

### Topical Cream

For *in vivo* treatment, LASSBio-1386 was used in a 1% w/w formulation in base cream. For a more homogeneous dilution in the base cream, the compound was weighed and dissolved in DMSO. Cream oil-in-water (O/A) emulsion was prepared according to the nonionic lotion II formula described in the Brazilian Pharmacopoeia Protocol, with some modifications. Phase 1 consisted of disodium EDTA (0.10% w/w) and purified water (88.90% w/w). Phase 2 consisted of nonionic self-emulsifying wax (cetostearyl alcohol, ethoxylated sorbitan monostearate) (9.00% w/w) and decyl oleate (2.00% w/w). Cream base emulsion was produced according to the general hot emulsification method. After weighing phases 1 and 2, they were separately heated to 75 °C (phase 1) and 80 °C (phase 2). After reaching working temperatures, both phases were combined under mechanical agitation. Stirring was maintained at a constant rate of 1,500 rpm until the emulsion was cooled to about 40 °C (Mota et al., 2020).

### *In vivo* Infection and Treatment

BALB/c mice were infected in the right ear dermis with 5 × 10^6^ *L. amazonensis* promastigotes at stationary phase in 10 μL of saline. After 2 weeks of infection, mice were treated daily with the LASSBio-1386 (1% cream) by topical route (covering the whole ear), for 5 weeks. To avoid the lack of stability, a cream with active principle was prepared each week. The development of lesion was monitored weekly using a digital caliper. The size of lesion was determined by the difference between the thickness of infected ear and thickness of uninfected contralateral ear. Parasite quantification was estimated by limiting dilution of lymph nodes of the infected mice treated or not treated with LASSBio-1386 (Guimaraes et al., 2009).

### Histopathological Analysis

Infected ears of the BALB/c mice were collected after the euthanasia of these animals, fixed in a solution of 10% formaldehyde for 48 h. Fragments of ear tissue were embedded in paraffin and sections (3–5  μm thick) were obtained and stained with conventional hematoxylin and eosin and analyzed by light microscopy (Guimaraes et al., 2009).

### Molecular Modeling Studies

#### Docking Studies

The compound was drawn in the Marvin Sketch 6.0 (ChemAxon, 2013) and had its coordinates optimized at the Sybyl-X 2.1.1 platform (Tripos, 2010) by conjugate gradient steps (convergence = 0.001 kcal/mol; interaction = 50,000) using Tripos force field, Gasteiger-Huckel charges (Gasteiger and Marsili, 1980) and implicit solvent (dielectric constant = 80.0). The crystallographic structure of *L. major* phosphodiesterase (*Lmj*PDE; PDB 2R8Q) was used for LASSBio-1386 docking calculations. Before of test, ions, co-crystallized ligands and water molecules not relevant to the system structure were removed. The protein binding site for molecular docking was centered on the 3-isobutyl-1-methylxanthine ligand (IBM) coordinates and the GOLD 5.7 software (Jones et al., 1997) was employed for calculations. The GOLD 5.7 settings and scoring function that allowed the lowest RMSD between docking and crystallographic IBM coordinates was employed for dock LASSBio-1386 in the *Lmj*PDE4. Visual inspection of the structures was performed with PyMOL 1.8 software (Schrodinger, 2015).

#### Molecular Dynamics Studies

The GROMACS 2019 software (Van Der Spoel et al., 2005) was employed for molecular dynamics (DM) simulations. The following parameters were used: time = 100 ns, 1 atm, 298 K, pH 7.0, GROMOS53A6 force field, electrostatic treatment of PME, 1.0 nm for non-covalent interactions, periodic boundary conditions - PBC, 1 ps writing steps, SPC/E solvency, octahedral space. To ensure electrical neutrality, counter ions were added to the system. Once these steps were completed, the system was energy minimized (steepest descent/conjugate gradient) until forces are lower than 30 kJ mol^−1^ nm^−1^. Following, a 1 ns simulation with position restrained on heavy atoms was performed (pre-equilibrium step) under DM simulation conditions, temperature set at 298 K and system pressure maintenance at 1 atm (constant NPT). In all calculations, to better simulate biological conditions, lysine and arginine residues were considered protonated, while HIS-85 will be kept deprotonated. The simulations under the mentioned conditions were performed in the *Lmj*PDE apo structure as well as in the *Lmj*PDE: LASSBio-1386. The ligand topologies for complexes simulations were built on the Automated Topology Builder (ATB) server (http://compbio.biosci.uq.edu.au/atb/). The initial coordinates of LASSBio-1386 in the *Lmj*PDE:IBM complex was obtained from docking. Representative structures of the *Lmj*PDE:LASSBio-1386 complex were extracted from DM simulations with the clustering algorithm (GROMOS) available in Gromacs 2019, with a cut-off value of 0.20 nm.

#### Estimation of Binding Free Energies

Besides molecular docking and molecular dynamics simulation studies, molecular mechanics/Poisson-Boltzmann surface area (MM-PBSA) were applied to determine the thermodynamical stability of *Lmj*PDEB1-LASSBio-1386 complex and also to inspect the contribution of each residue of the binding pocket. The MM-PBSA were calculated through a script-based *g_mmpbsa* tool (Kumari et al., 2014). This method calculates the binding energy (∆E_binding_) which represents the average two energetic terms: potential energy in the vacuum (ΔE_MM_) and the free solvation energy (∆G_solvation_), as described in equation.

$$\Delta E_{\text{binding}}=\Delta E_{\text{MM}}+\Delta G_{\text{solvation}}$$ (1)

The molecular mechanic (MM) energy term (ΔE_MM_) is calculated from an electrostatic (ΔE_elec_) and van der Waals (ΔE_*vdW*_) interactions components based on the molecular mechanics force-field parameters (Kumari et al., 2014). The solvation energy is computed from polar (ΔG_pol_), using the Poisson–Boltzmann (PB) equation (Honig and Nicholls, 1995; Srinivasan et al., 1998; Baker et al., 2001), and nonpolar solvation energy (ΔG_nonpol_), estimated from the solvent-accessible surface area (SASA) including repulsive and attractive forces between solute and solvent that are generated by cavity formation and van der Waals interactions (Kumari et al., 2014). To decompose the binding energy, at first ΔE_MM_, ΔG_pol_ and ΔG_nonpol_ are separately calculated for each residue and were then summed up to obtain the contribution of each residue to the binding energy (Kumari et al., 2014).

The energy components E_MM_, G_pol_ and G_nonpol_ of LmjPDEB1 (Apo) and LmjPDEB1-LASSBio-1386 complex were calculated for 700 snapshots extracted every 0.1 ns from the production trajectories from 30 to 100 ns. E_MM_ was calculated using the LJ and Coulomb potential. To calculate G_pol_, a box was generated using the extreme coordinates of the molecular complex in each dimension. The box was then expanded in each dimension by 1.5-fold to obtain a coarse-grid box (cfac = 1.5). A finer grid-box is then placed within the coarse grid-box extending 5 Å (fadd = 5) from the complex’s extreme coordinates in each direction. An ionic strength of 0.150 M NaCl with radii of 0.95 and 1.81 Å for sodium and chloride ions respectively was used during all G_*pol*_ calculations. The values for the vacuum (vdie), solvent (sdie) and solute (pdie) dielectric constants were taken as 1, 80 and 2, respectively. The solvent radius was set to 1.4 Å and temperature, 303 K. The linear PB equation was solved using APBS program (Holst & Saied, 1993; Holst and Saied, 1995; Bank and Holst, 2000; Jurrus et al., 2018). G_nonpol_ was calculated using solvent accessible surface area (SASA) nonpolar models using the surface tension (gamma) 0.0226,778 kJ/(mol A2) and probe radius 1.4 Å.

### Statistical Analysis

One-way analysis of variance and Newman-Keuls multiple comparison tests were employed by using Graph Pad Prism version 5.01 (Graph Pad Software, San Diego, CA, United States). Differences were considered significant when the values of *p* were <0.05 (Guimarães et al., 2018).

## Results

### Cytotoxicity Evaluation

LASSBio-1386 presented a CC_50_ value of 74.1 μM after 24 h of incubation, being less cytotoxic than amphotericin B (CC_50_ = 39.4 μM) and gentian violet (CC_50_ = 0.5 μM), a known cytotoxic drug.

### Antileishmanial Activity of LASSBio-1386 Against Promastigote and Intracellular Parasites

The activity of LASSBio-1386 was first evaluated against promastigotes of *L. amazonensis*. LASSBio-1386 was tested at six different concentrations, ranging from 0.625 to 20 µM. After 72 h of incubation, LASSBio-1386 inhibited promastigote proliferation with an EC_50_ of 2.4 ± 0.48 µM, while the reference drug amphotericin B presented an EC_50_ of 0.03 ± 0.006 µM. Regarding the selectivity index (SI), LASSBio-1386 exhibited a selectivity of 30.8 and amphotericin B presented a SI of 437.7 (Table 1).

**TABLE 1:** Cytotoxicity evaluation (CC_50_), half maximal effective concentration for 50% of promastigotes and intracellular parasites forms of (EC_50_) and selectivity index (SI).

Compounds	Mφ J774	*L. amazonensis*			
		Promastigotes		Intracellular parasites	
	CC_50_ ± S.D. (µM)	EC_50_ ± S.D. (µM)	SI	EC_50_ ± S.D. (µM)	SI
LASSBio-1386	74.1 ± 2.9	2.4 ± 0.48	30.8	9.42 ± 0.69	7.8
Amphotericin B	39.4 ± 1.4	0.09 ± 0.02	437.7	0.055 ± 0.021	716.3
Gentian violet	0.5 ± 0.09	N.D.	N.D.	N.D.	N.D.

CC_50_ values and EC_50_ values for intracellular parasites were determined after 24 h of incubation. EC_50_ values for promastigotes were determined after 72 h of incubation. N.D. = not determined; S.D. = Standard deviation.

**FIGURE 2:**
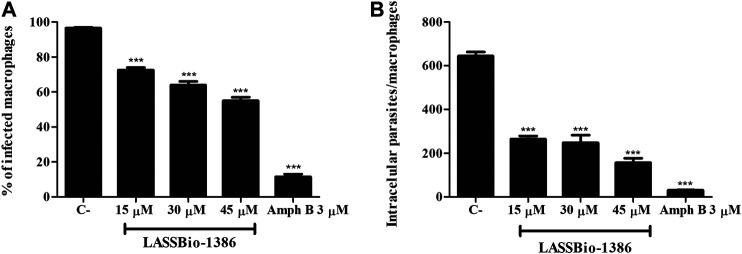
*In vitro* effects of LASSBio-1386 against intracellular forms of *L. amazonensis*. J774 macrophages were infected with *L. amazonensis* promastigotes in stationary phase (10:1) and were incubated or not with LASSBio-1386 (15, 30 and 45 µM) for 24 h **(A and B)**. Amphotericin B was used as positive control at concentration of 3 µM. The percentage of infection and number of intracellular parasites/100 macrophages were determined after 24 h of incubation with LASSBio-1386. C-: untreated control; ***p < 0.001, compared to untreated group.

Next, J774 macrophages were infected with *L. amazonensis* promastigotes and incubated with the LASSBio-1386 after 4 h of infection. Incubation with LASSBio-1386 (15, 30 or 45 µM) promoted a significant decrease in the percentage of infected cells and the number of intracellular parasites 24 h after incubation (Figures 2A,B). LASSBio-1386 had an EC_50_ value of 9.42 ± 0.64 µM against *L. amazonensis* intracellular forms (Table 1). Amphotericin B decreased the number of infected cells and intracellular parasites by more than 80% (Figures 2A,B) and presented an EC_50_ value of 0.055 ± 0.021 (Table 1). Regarding the selectivity index (SI), LASSBio-1386 exhibited a selectivity of 7.8 and amphotericin B presented a SI of 716.3 (Table 1).

### Mechanism Action Studies

**FIGURE 3:**
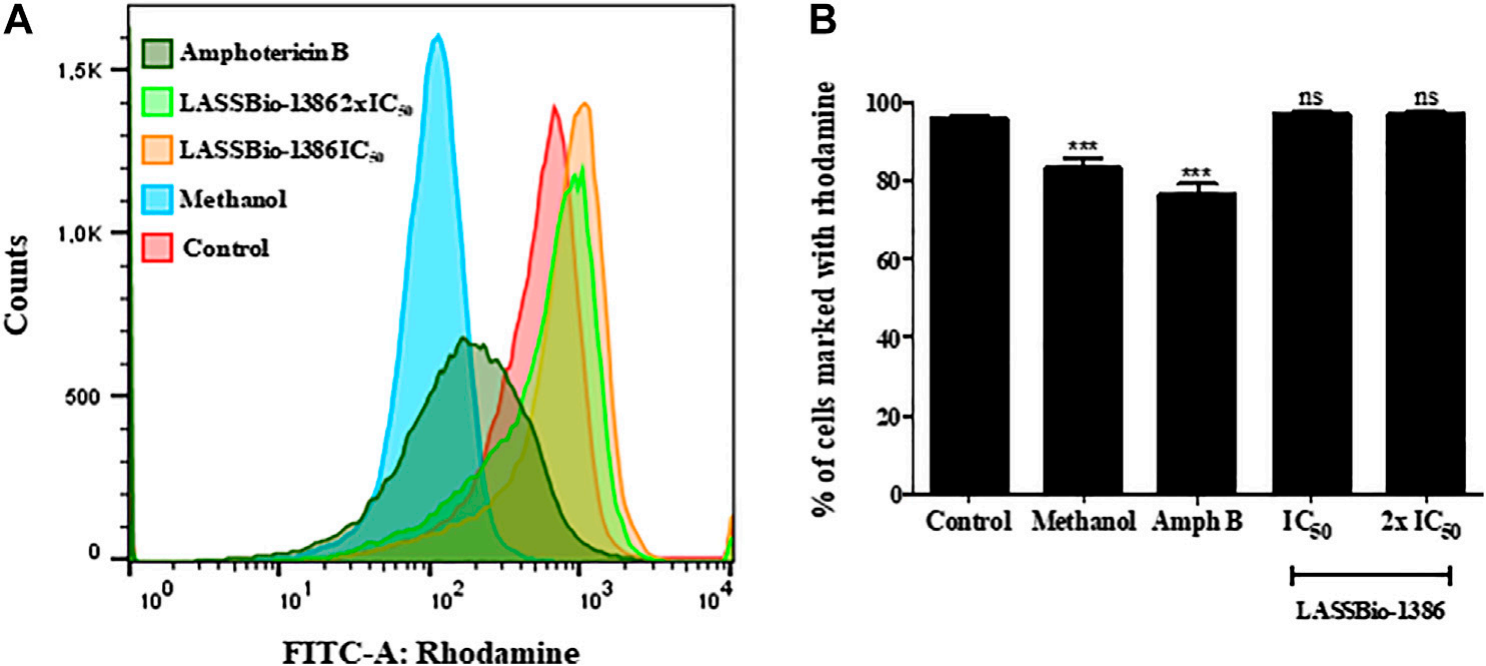
Mitochondrial membrane potential of *L. amazonensis* promastigotes incubated with LASSBio-1386. Promastigotes of *L. amazonensis* (1 × 10^6^) were incubated or not with LASSBio-1386 (at concentrations of EC_50_ and 2x EC_50_ for promastigote forms). After 72 h of incubation, parasites were marked with rhodamine 123 **(A)** and the intensity of rhodamine 123 was not significantly altered by incubation with LASSBio-1386 at EC_50_ and 2xEC_50_ concentrations **(B)**. Methanol was the positive control. The samples were acquired in a LSRFortessa flow cytometer and analyzed by FlowJo software (50,000 events were collected and analyzed). ***p < 0.001, compared to untreated group.

To investigate the mechanisms by which this compound affects the parasite, we first investigated possible alterations in the mitochondrial membrane potential of *L. amazonensis* promastigotes incubated with LASSBio-1386. The intensity of rhodamine 123, a fluorescent dye sequestered by active mitochondria, was not significantly altered by incubation with LASSBio-1386 at EC_50_ and 2xEC_50_ concentrations (Figure 3). In contrast, methanol and amphotericin B, two known agents able to induce mitochondrial alterations, reduced the intensity of the rhodamine 123. This suggests that LASSBio-1386 inhibits *L. amazonensis* growth without affecting the parasite’s mitochondrial function.

**FIGURE 4:**
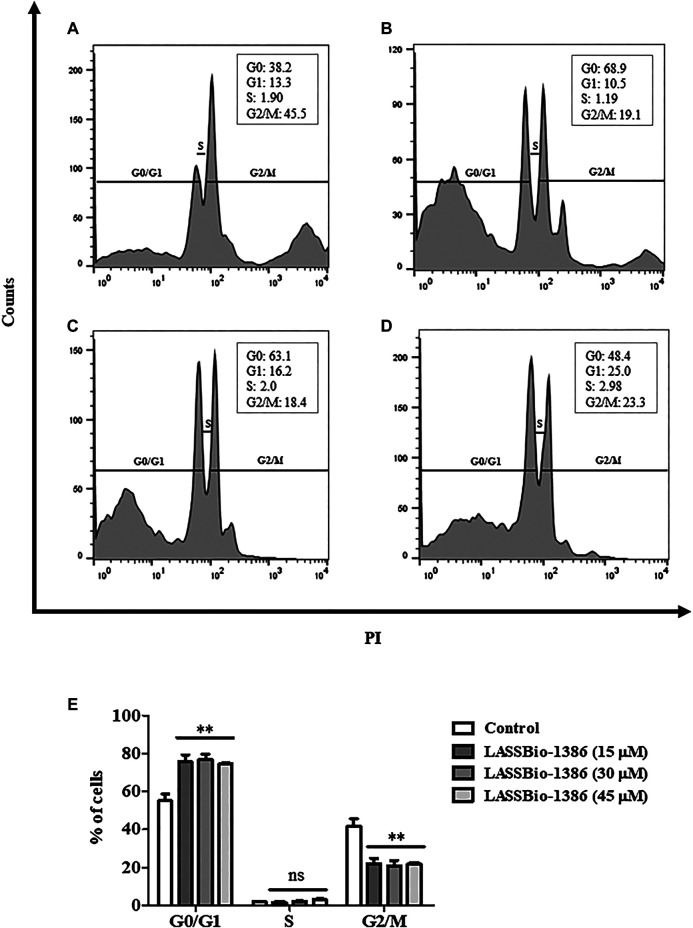
Analysis of the cell cycle using propidium iodide by flow cytometry. Promastigotes of *L. amazonensis* (1 × 10^7^) were incubated or not with different concentrations of LASSBio-1386 for 48 h. After incubation, parasites were marked with propidium iodide. The samples were acquired in a LSRFortessa flow cytometer and analyzed by FlowJo software. A significant increase in population of cells in G0/G1 and a significant decrease in population of cells in G2/M **(E)** were observed with 15 **(B)**, 30 **(C)** and 45 µM **(D)** concentrations of LASSBio-1386, compared to untreated control **(A)**. C-: untreated control; **p < 0.01, compared to untreated group.

Next, quantification of nuclear DNA of promastigotes, which indicates the phases of the cell cycle, was analyzed by flow cytometry using propidium iodide as a fluorogenic agent. The cell cycle analysis indicated that incubation with LASSBio-1386 at 15, 30 and 45 μM caused cell cycle arrest in the G0/G1 phase of *L. amazonensis* promastigotes, compared to untreated controls. Also were observed a significant decrease in population of cells in G2/M (Figure 4).

**FIGURE 5:**
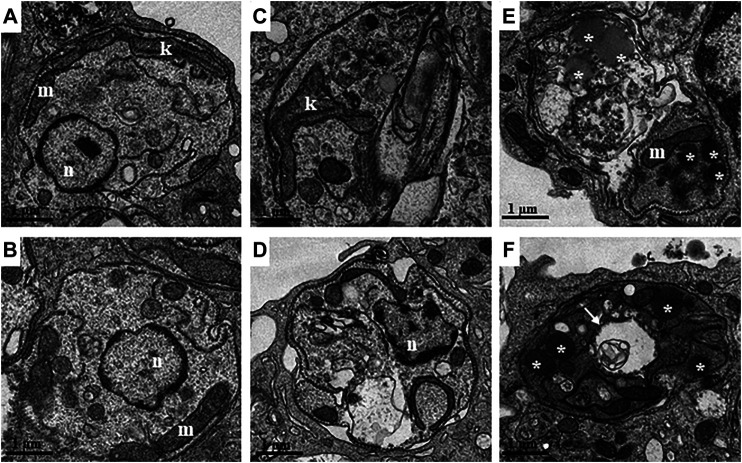
Transmission electron microscopy (TEM) analysis of intracellular parasites of *L. amazonensis* incubated with LASSBio-1386. Macrophages infected with *L. amazonensis* were incubated with LASSBio-1386 (30 and 45 μM) for 24 h. After incubation, cells were fixed, post-fixed and embedded in polybed resin. Ultrafine sections were contrasted and observed under a JEOL TEM-1320 transmission electron microscope. **(A and B)** Untreated intracellular parasites showing organized cytoplasm and organelles with normal morphology. **(C and D)** Intracellular parasites incubated with LASSBio-1386 at a concentration of 30 μM with increased kinetoplast and cytoplasmic disorganization. **(E and F)** Parasites incubated with LASSBio-1386 at a concentration of 45 μM showing cytoplasmic degradation, lipid inclusions and autophagosomes (white arrow). n: nucleus; m: mitochondria; k: kinetoplast; *: lipid inclusions.

Ultrastructural analysis was also performed in intracellular parasites incubated with LASSBio-1386 by transmission electron microscopy. While untreated intracellular parasites showed organized cytoplasm and organelles with preserved morphology (Figures 5A,B), parasites from cultures incubated with LASSBio-1386 at 30 μM presented kinetoplast swelling (Figure 5C) and disorganization of the cytoplasm such as folds in the cytoplasmic membrane and change in the shape of the cell (Figure 5D). Moreover, parasites from cultures incubated with 45 μM of LASSBio-1386 also presented cytoplasmic degradation, presence of lipid inclusions and autophagosomes (Figures 5E,F).

### *In vivo* Effects of LASSBio-1386 on Experimental Model of Cutaneous Leishmaniasis

**FIGURE 6:**
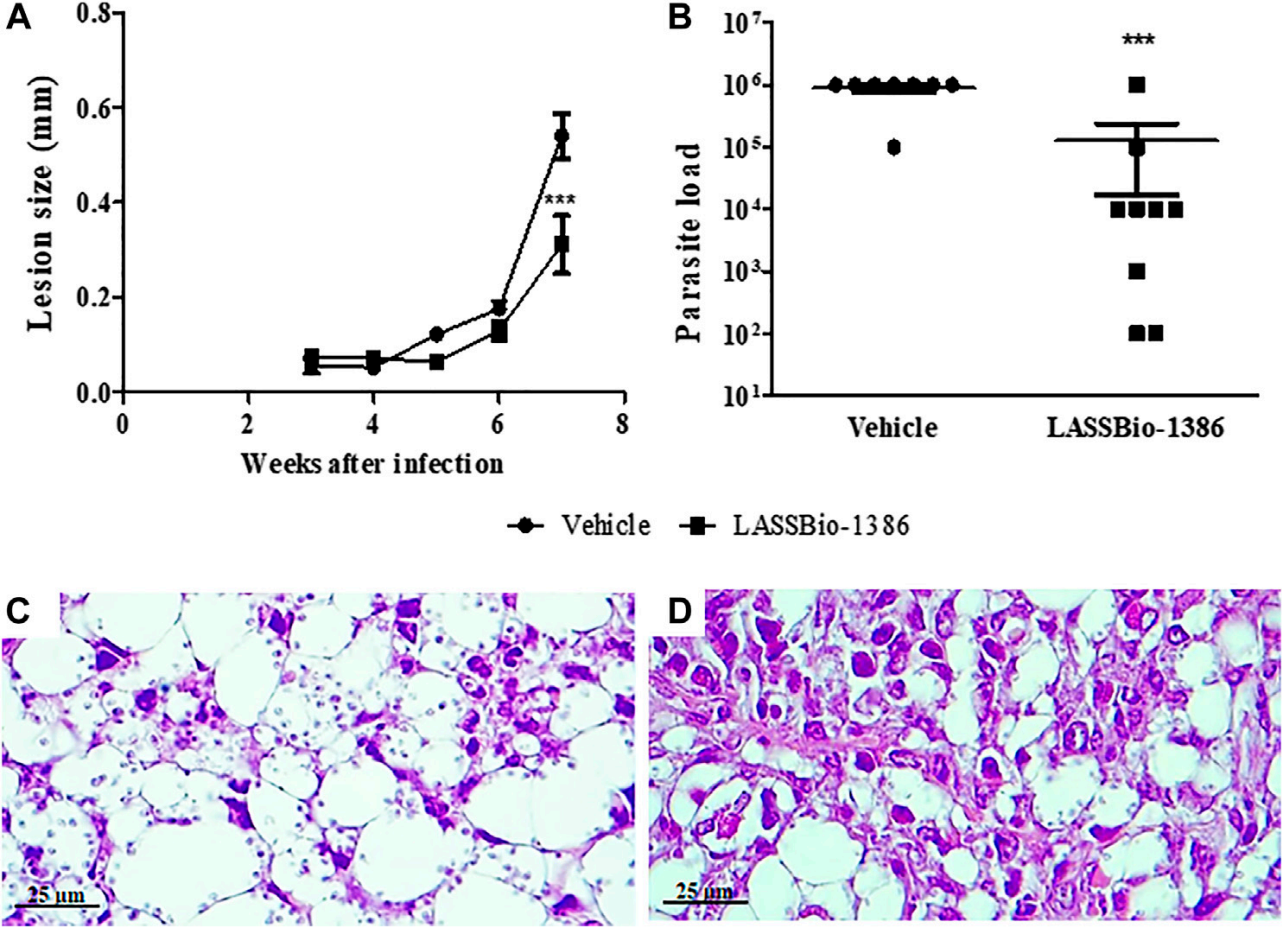
*In vivo* activity of LASSBio-1386. BALB/c mice were infected in the ear dermis with promastigotes of *L. amazonensis* (5 × 10^6^). After 2 weeks of infection, mice were untreated or treated daily topically with LASSBio-1386 for 5 weeks. **(A)** Lesion size of BALB/c mice untreated (vehicle) or treated was determined by the difference between the thickness of infected and uninfected ear. **(B)** Parasite load was estimated by limiting dilution of lymph nodes of infected mice untreated (vehicle) or treated with LASSBio-1386. A significant decrease in parasitic load was observed in BALB/c mice treated topically with LASSBio-1386. ***p < 0.001, compared to untreated group. After 5 weeks of treatment, infected ears of BALB/c mice were removed and fixed with formaldehyde solution (10%). Tissue sections (3–5  μm thick) were stained by hematoxylin and eosin and observed under light microscopy (Scale bar = 25 µm). **(C)** Infected mice untreated (vehicle). **(D)** Infected mice treated with LASSBio-1386.

To evaluate the *in vivo* effects of LASSBio-1386 treatment in cutaneous leishmaniasis, BALB/c mice were infected with *L. amazonensis* in the ear dermis and treated daily with the compound (1% topical formulation) or vehicle (group control treated with the base cream without the active principle), starting 2 weeks after infection. LASSBio-1386 treatment significantly reduced the lesion size when compared with vehicle-treated mice at 5 weeks after infection (Figure 6A). Importantly, LASSBio-1386 treatment also caused a significant reduction in the parasite number in the draining lymph nodes, compared with vehicle-treated mice (Figure 6B). Moreover, histopathological analysis showed that lesions from vehicle-treated animals presented predominantly an extensive and monomorphic collection of many vacuolated and parasitized macrophages (Figure 6C). Lymphocytes and others inflammatory cells were less frequent (Table 2). In contrast, lesions of LASSBio-1386-treated mice had a mixed inflammatory infiltrate, containing vacuolated and non-vacuolated macrophages with reduced parasite numbers, polymorphonuclear cells and lymphocytes (Table 2 and Figure 6D).

**TABLE 2:** Histopathological analysis of mice infected by *L. amazonensis* and treated or no with LASSBio-1386.

	Experimental groups	
	Vehicle (*n* = 5)	LASSBio-1386 (*n* = 5)
Vacuolated macrophages	+++++ (4/5 mice)	+++++ (2/5 mice)
	+++ (1/5 mice)	+++ (3/5 mice)
Lymphocytes	+++ (2/5 mice)	+++++ (1/5 mice)
	+ (3/5 mice)	+++ (4/5 mice)
Polimorphonuclear cells	+++ (1/5 mice)	+++ (3/5 mice)
	+ (4/5 mice)	+ (2/5 mice)
Parasitism	+++++ (5/5 mice)	+++ (5/5 mice)

Intensity: + = minimum; +++ = intermediary; +++++ = accentuated. The number of mice per group displaying each feature are indicated in parenthesis.

### Molecular Modeling Studies

**FIGURE 7:**
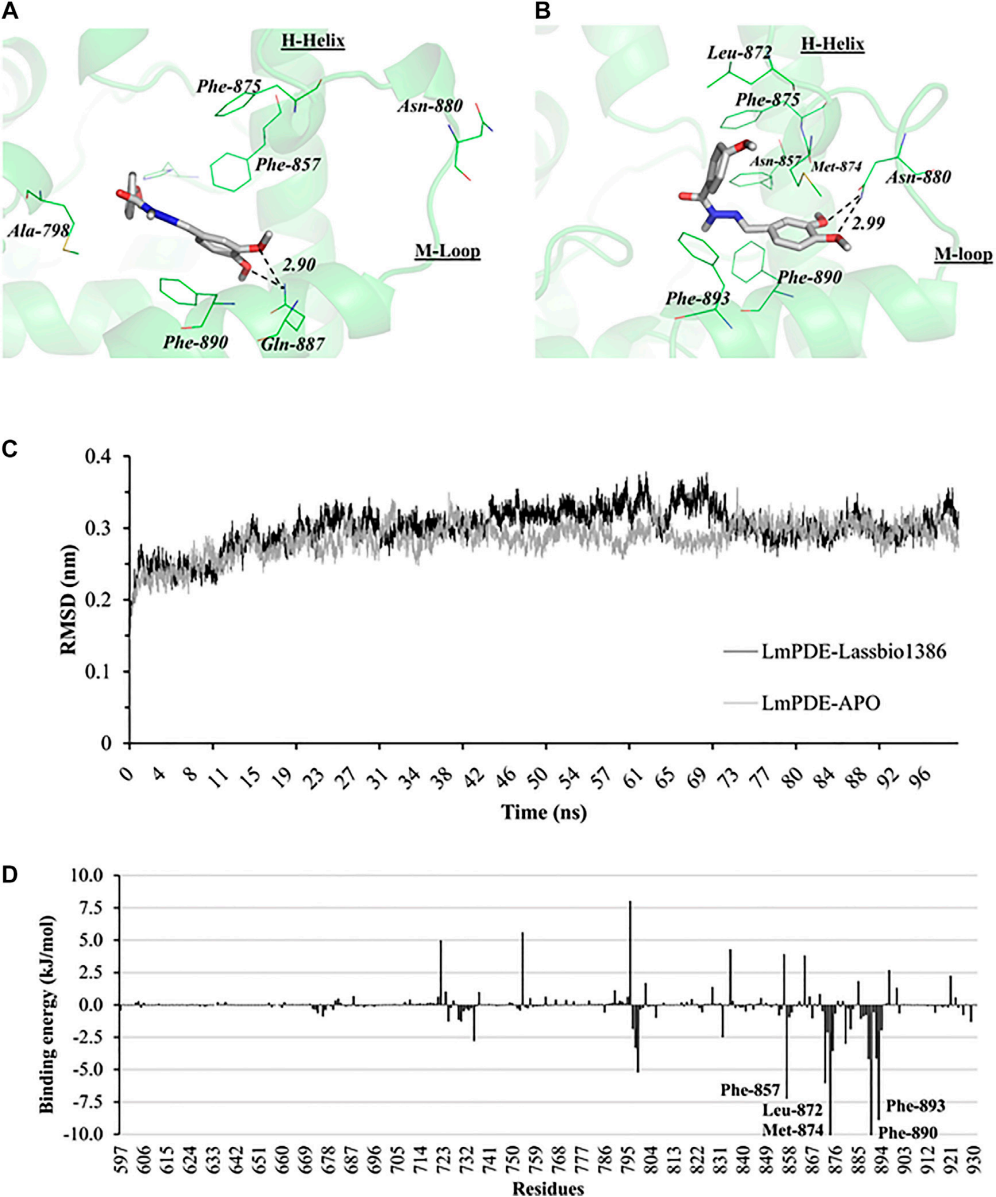
*In silico* activity of LASSBio-1386. **(A)** Putative binding profile of LASSBio-1386 toward *Lmj*PDEB1 catalytic binding site according the GOLD 5.3 best ranked pose. **(B)** Representative conformation of LASSBio-1386 according to MD simulations (100 ns). The *Lmj*PDEB1 binding site residues are depicted in line, whereas LASSBio-1386 is depicted in stick model. The hydrogen bonds are displayed as dashed line and distances are measured in angstroms. During 97% of the trajectory time, a H bond forms between LASSBio-1386 and Asn-880. **(C)** Root-mean-square deviation calculated on Cα positions over trajectory with respect to initial structures for *Lmj*PDEB1 in APO form and complexed with LASSBio-1386 **(D)** Residue contributions to the binding energy of *Lmj*PDEB1-LASSBio-1386 complex. Residues with ΔEbinding > ± 5 kJ/mol were highlighted.

*In silico* methods, such docking and molecular dynamics, were applied to understand the LASSBio-1386 and *Leishmania* phosphodiesterase (*Lmj*PDEB1) structural features related to inhibition and provide a detailed description of these intermolecular interaction profiles. At first, the validation step performed by redocking the IBM in the *Lmj*PDEB1 and comparison to IBM crystallographic pose (Supplementary Figure S2) suggested that the docking parameters are reliable (RMSD redocking value for IBM docked vs. IBM crystallographic poses = 0.6 Å). Therefore, these parameters were employed to dock LASSBio-1386 in the *Lmj*PDEB1 (Figure 7). After calculation, the best docking pose of LASSBio-1386 at the *Lmj*PDEB1 catalytic site suggests the presence of *π*-stacking interactions involving the aromatic rings of Phe-857 and Phe-890 and hydrogen bonding (H-bond) with Gln-887 (Figure 7A).

Following the *Lmj*PDEB1-LASSBio-1386 better docked complex was submitted to the MD routine. The system evolution along MD simulation demonstrates its stabilization (average RMSD_30–100 ns_ = 0.36 ± 0.06 nm) from 30 ns onwards (Figure 7C).

To understand the biophysical basis of the molecular interactions of LASSBio-1386 and *LmjPDE*B1, a MM-PBSA scheme was employed. It provided the binding energy (ΔE_*binding*_) of *LmjPDE*B1-LASSBio-1386 complex (−118.71 ± 0.70 kJ/mol) and the summary of binding components as follows. Most of such low binding energy has a high contribution of molecular mechanic components ΔE_vdW_ (−182.2 ± 0.45 kJ/mol) and ΔE_elec_ (−16.44 ± 0.55 kJ/mol), followed by non-polar solvation energy ΔG_nonpol_ (−17.71 ± 0.04 kJ/mol), although unfavorable compensated by polar solvation energy ΔG_pol_ (97.48 ± 0.37 kJ/mol).

In order to evaluate which *LmjPDE*B1 residues contribute better to LASSBio-1386 interactions, the binding energy was decomposed by each residue (Supplementary Table S1). For the analysis clarity, we just selected residues near the ligand (<5 Å) during the MD simulation and which participate actively in complex stabilization (ΔE_binding_ > ± 5 kJ/mol) as shown in Figure 7D.

The *Lmj*PDEB1 ΔE_binding_ residues analysis suggests that Phe-857 (−7.23 ± 0.06 kJ/mol), Leu-872 (−6.02 ± 0.05 kJ/mol), Met-874 (−10.94 ± 0.09 kJ/mol), Phe-890 (−10.45 ± 0.07 kJ/mol) and Phe-893 (−8.88 ± 0.07 kJ/mol) presented favorable contributions for LASSBio-1386 affinity and selectivity on LmjPDEB1 binding site (See Supplementary Table S1). In order to evaluate these interactions with LASSBio-1386, the complex representative structure was extracted (Figure 7B). This analysis demonstrated that LASSBio-1386 maintained the same interactions with Phe857 and 890 at the catalytic site previously suggested by our docking results (Figure 7A). The Phe-893 and Leu-872 residues also makes *π*-stacking interactions and hydrophobic contact with *p*-methoxy phenyl moiety of LASSBio-1386 while Met-874 residue made a favorable hydrophobic interaction with both dimethoxy phenyl ring and *p*-methoxy phenyl moiety.

This new interaction mode is related to conformational change on Helix-H and loop M (Figures 7A,B) which also allows the dimethoxy phenyl moiety interacts through hydrogen bond (H-bond) with the Asn-880 residue. This interaction is high prevalent (>97% of the molecular dynamic simulation time) (Supplementary Figure S2) and presented a favorable binding energy (ΔE_binding_ = −2.98 ± 0.09 kJ/mol, Supplementary Table S1), indicating that this H-bond is important for ligand affinity. On the other hand, the H-bond analysis also suggested additional interaction between LASSBio-1386 and His-800 during >65% of the simulation time (Supplementary Table S2). However, the very low binding energy contribution (ΔE_binding_ = −0.34 ± 0.04 kJ/mol) supports that His-800 interaction could not be important for ligand affinity.

## Discussion

From the medicinal chemistry perspective, *N*-acylhydrazones are an important class of organic compounds that can interact with various bioreceptors. These molecules can establish hydrogen bonds as an electron donor or acceptor and adopt several favorable conformational orientations due to the characteristics of their chemical structure (Fraga and Barreiro, 2006). Among these compounds, the methylated NAH derivative (E)-N′-(3,4-dimethoxybenzylidene)-4-methoxy-N-methylbenzohydrazide (LASSBio-1386) has been identified as a potent inhibitor of PDE-4 activity (Kümmerle et al., 2012; Guimarães et al., 2018). Given the high structural similarity shared between human and protozoan PDEs, this allowed us to hypothesize that LASSBio-1386 has activity against species from the Leishmania genus (Seebeck et al., 2011). Thus, in the present work, we investigated the antileishmanial activity of LASSBio-1386 *in vitro* and *in vivo,* and evaluated *in silico* its interaction with leishmania PDE.

LASSBio-1386 showed activity against promastigote and intracellular forms of *L. amazonensis* with low toxicity to mammalian cells, showing selectivity to *L. amazonensis*. Current therapy against leishmaniasis based in amphotericin B and Glucantime^®^ has several side effects due to their high toxicity (Mendonça et al., 2018). Thus, it is important to look for new compounds more effective in leishmaniasis treatment and with a better safety profile. Despite the lower toxicity of LASSBio-1386, amphotericin B had a better selectivity index due to its higher potency in infected cells. Selectivity index (SI) value indicates selectivity of the sample to the cell lines tested. Any sample which has SI value higher than three will be considered to have high selectivity (Prayong et al., 2008). Right before this cutoff point, LASSBio-1386 showed high selectivity. However, amphotericin B has an elevated cost, is highly toxic and its use requires hospitalization of patients. Furthermore, amphotericin B is a drug with difficult structural changes in the molecule (Chakravarty & Sundar, 2019), whereas LASSBio-1386 is a prototype whose selectivity can be increased with conformational alterations (Kümmerle et al., 2012).

The study of mechanisms of action provides vital information of drug development process and possible biochemical targets (Schenone et al., 2013). The proper maintenance of the mitochondrial membrane potential is essential for cell survival, since it it associated to the function of mitochondria. Furthermore, trypanosomatids have a single mitochondrion, which makes this organelle a potential target for drugs (Vannier-Santos et al., 2012; Aliança et al., 2017). In the present study, different than amphotericin B, LASSBio-1386 incubation did not result in depolarization of mitochondrial membrane potential of the parasite, suggesting that the compound cause the death of parasites by another biochemical pathway. We also evaluated the influence of the LASSBio-1386 on the different phases of the cell cycle. Cell analysis exhibited a increase in the G0/G1 phase of treated promastigotes. The high peak of G0/G1 phase indicates DNA fragmentation and suggests the apoptotic-like cell death (Shadab et al., 2017; Stroppa et al., 2017). In a recent study of our group, LASSBio-1386 inhibited lymphocyte proliferation, triggering a cell cycle arrest in G0/G1 phase (Guimarães et al., 2018).

Ultrastructural changes of the intracellular parasites also reinforce a direct action of this derivative in the parasite. Among the observed alterations, there is the accumulation of lipid inclusions. Some authors suggest that the increase in the number of these inclusions may occur as a result of the accumulation of drug action and due to inhibition of lipid metabolism (Rodrigues et al., 2007; De Medeiros et al., 2011). The presence of structures suggestive of autophagic vacuoles may indicate the induction of autophagy after treatment with LASSBio-1386. The autophagy-related proteins may facilitate the elimination of invading pathogens (Randow and Munz, 2012; Deretic et al., 2013). In fact, stress conditions, such as drug treatments, are able to induce an autophagic phenotype that is seen through the appearance of myelin-like structures, multivesicular bodies and an increase in the number of autophagosomes (Bera et al., 2003; Delgado et al., 2008; Menna-Barreto et al., 2009b; Koh et al., 2015; Dos Anjos et al., 2016). Myelin-like structures and concentric membrane structures, are the most commonly found evidence of autophagy in parasites under stress condition. The term “Autophagic cell death” has been used when the homeostatic control is lost and autophagy is exacerbated to degrade damaged structures, macromolecules or organelles (Menna-Barreto et al., 2009a). Other compounds with antileishmanial activity were also able to induce autophagy in *Leishmania* (Mendes et al., 2016; Scariot et al., 2017; Costa et al., 2019). Additionally, the combination therapy of miltefosine and a semi-synthetic thiosemicarbazone caused a significant increase in the number of autophagic vacuoles (Scariot et al., 2017).

The *in vivo* activity of LASSBio-1386 was investigated using BALB/c mice, which are highly susceptible to *Leishmania* infection (Afrin et al., 2019). Treatment by topical route with LASSBio-1386 decreased the size of the lesion and the parasite load. In fact, some compounds from the hydrazone class, such as dialkyl phosphohydrazone, reduced the lesion size in the ear infection model, and one compound of this class reduced the parasite load (Matta et al., 2015). The beneficial effect of LASSBio-1386 *in vivo* may result from both the antileishmanial activity, leading to the observed reduction of *Leishmania* parasites *in vivo*, as well as to a modulation of the immune response. The inflammatory response during cutaneous leishmaniasis also has a significant role in tissue damage in the lesions. In a previous study from our group, the anti-inflammatory effect of LASSBio-1386 was evaluated (Guimarães et al., 2018). LASSBio-1386 caused a reduction of lymphocyte proliferation in a concentration-dependent manner, reduced the production of IL-2 and IFNγ by activated splenocytes and the levels of NO and TNF-α, produced by macrophages upon activation with LPS and IFNγ, being associated with a down-regulation of NF-κB pathway (Guimarães et al., 2018). Thus, in addition to the antileishmanial activity, the anti-inflammatory properties of LASSBio-1386 may also be relevant for the healing process in cutaneous leishmaniasis lesions.

As previously mentioned, LASSBio-1386 affected the *Leishmania* cell cycle and therefore shed light on cAMP phosphodiesterase (*Lmj*PDEB1) as a possible macromolecular target. In fact, the PDEB1 enzyme is essential for trypanosomal proliferation (Ekholm et al., 1999) and its knockdown cause severe cell cycle damages and cell death, both *in vitro* and *in vivo* (Oberholzer et al., 2007; Orrling et al., 2012). Together, these facts reinforce our initial hypothesis that LASSBio-1386 is acting against *L. major* and *L. amazonensis* through their PDE modulation.

In order to analyze the structural requirements for *Lmj*PDE1 inhibition, the docking assay was done and suggested that LASSBio-1386 dimethoxy phenyl core is stabilized by Phe-857 and 890 residues of *Lmj*PDEB1 through *π*-stacking interactions. Similarly, the structurally conserved residues of *Hs*PDE4D (Phe-340 and Phe-372) interacted with the same dimethoxy phenyl portion of *N*-acylhydrazones derivative (Kümmerle et al., 2012) (See Supplementary Figure S1). For *Hs*PDE3B, equivalent residues (Phe-959 and Phe-991) interacted with phenyl and pyridazinone moieties of zardaverine in its crystallographic structure (Card et al., 2004). The docking also showed dimethoxy phenyl portion of the LASSBio-1386 made hydrogen bonds with Gln-887 residue of *LmjPDE*B1 resembling the interaction involving dimethoxy phenyl portion of *N*-acylhydrazones derivative and Gln-369 from *Hs*PDE4D (Kümmerle et al., 2012).

However, as the docking protocol did not explore the enzyme flexibility and neither mimics its induced-fit behavior caused by the presence of the ligand, the LASSBio-1386-*Lmj*PDEB1 complex obtained by docking was submitted to a 100 ns MD, a simulation with time long enough to allow side chains accommodation. The complex was stable throughout productive phase of simulation (30–100 ns, Figure 7A) which allowed us to compute the binding energy of *Lmj*PDEB1-LASSBio-1386 complex through MM-PBSA protocol. The high contribution of ΔE_vdW_ for complex free energy (ΔE_binding_) suggest that non-polar and *π*-stacking interactions are crucial for ligand binding. Nonpolar groups can establish van der Waals contacts with the target that result in small enthalpic gains, whose magnitude depends on the degree of shape complementarity between compound and target (Kawasaki and Freire, 2011). Indeed, the subsite explored for LASSBio-1386 binding are surrounded for several aromatic and non-polar residues such as phenylalanine (857, 875, 890 and 893), Tyr-858, Met-874, Val-894 and Ala-798, that may be explain the energy behavior of complex.

The binding energy contribution per residue and the 3D analysis of *Lmj*PDEB1-LASSBio-1386 representative structure reinforce that Phe-857 and Phe-890 have favored the ligand binding (see Figures 7B,D) with the same interaction pattern described for *N*-acylhydrazones derivatives against *Hs*PDE4D (Kümmerle et al., 2012). This combined analysis also shows that Phe-893 residue presented a great favorable contribution energy for LASSBio-1386 affinity. In humans, the structural similar residues, Tyr-375 and His-994, present on *Hs*PDE4D (Supplementary Figure S1) and *Hs*PDE3B may perform an equivalent interaction.

Our MD simulation also revealed the Asn-880 residue H-bonding seems to be important for complex stability. This interaction presented significant electrostatic contribution (ΔE_elec_ = −14.98 ± 0.1 kJ/mol) despite of polar solvation (ΔG_pol_ = 12.49 ± 0.08 kJ/mol), resulting on favorable binding enthalpy_*.*_ This highlighted the role of Asn-880 on LASSBio-1386 affinity, since generally, only strong hydrogen bonds are able to overcome the unfavorable desolvation enthalpy of polar groups (Kawasaki and Freire, 2011).

It is noteworthy that the interaction with Asn-880 requires an approximation of the M-loop and H-Helix (Figures 7C,D) which is related to a sub-pocket exclusively accessible for *Leishmania* PDEs (Wang et al., 2007). This conformational change allows additional favorable interaction with Phe-875 (ΔE_binding_ = −3.54 ± 0.05 kJ/mol, Supplementary Table S1). These results obtained from our MD simulation reinforce the importance of these region for PDE inhibitor design (Huai et al., 2004; Wang et al., 2006; Ke and Wang, 2007).

Therefore, the new interaction pattern revealed by our computational studies could be explored in a future for the development of more selective and potent compounds against *Leishmania* parasites.

## Conclusion

Taken together, this is the first report demonstrating that LASSBio-1386 has a potential antileishmanial activity *in vitro* and *in vivo*. This compound represents a promising molecule due to its low cytotoxicity, activity against parasitic proliferation, macrophage infection and treatment of infection *in vivo*. *In silico* analysis suggest structural features that could be explored to improve the activity and selectivity against human PDE. Thus, LASSBio-1386 can be an important alternative for the development of new therapeutic agents with antileishmanial activity.

## Data Availability Statement

The raw data supporting the conclusions of this article will be made available by the authors, without undue reservation, to any qualified researcher.

## Ethics Statement

The animal study was reviewed and approved by Institutional Animal Care and Use Committee, Ethics Committee for Animal Experimentation of FIOCRUZ (CEUA/FIOCRUZ Permit Number: L-IGM-004/2019).

## Author Contributions

EG and MS conceptualized the project. TS and EB were responsible for synthesis of compounds. DS and JT conducted the biological assays. DM contributed *in vitro* assays. AT, SP, and HF conducted the *in silico* assays. All authors co-wrote the first draft of the manuscript and proofread the submitted manuscript.

## Funding

This work was supported by grants from CNPq (grant number 562655/2010-7) and PRONEX (grant number 0002/2014).

## Conflict of Interest

The authors declare that the research was conducted in the absence of any commercial or financial relationships that could be construed as a potential conflict of interest.
