# Supplementary material for: In Vitro, In Vivo and In Silico Effectiveness of LASSBio-1386, an N-Acyl Hydrazone Derivative Phosphodiesterase-4 Inhibitor, Against Leishmania amazonensis
Source: Front Pharmacol. 2020 Dec 16;11:590544. doi: 10.3389/fphar.2020.590544 (PMC7772393; doi:10.3389/fphar.2020.590544)
Supplement: Supplementary file 1 [file datasheet1.docx]

Supplementary Material

## Supplementary Figures


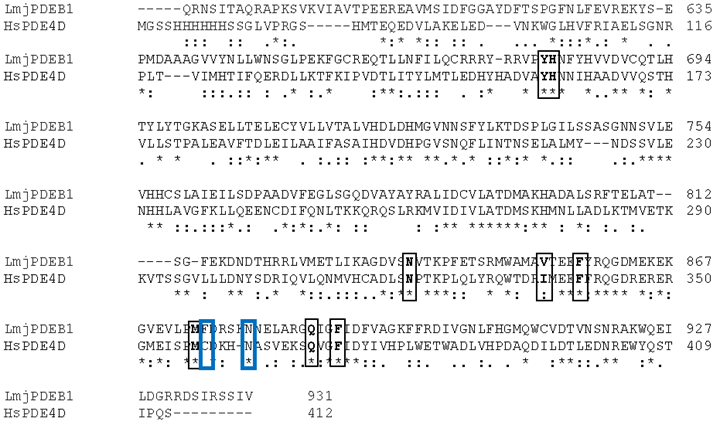


**Figure 1S.** Sequence alignment between *Homo sapiens* PDE4 (*Hs*PDE4) and *Leishmania major* PDEB1 (*Lmj*PDEB1) generated on the Clustal Omega server (SIEVERS et al., 2011). Global Sequential Identity and similarity = 29% and 51%; Catalytic site local Sequential Identity = 87% ("*" = Identical, ":" = Similar, "." = Not conserved, "-" = absent). Catalytic site residues of both enzymes are highlighted in black rectangles and new *Lmj*PDEB1 residues that interact with LASSBio-1386 after dynamic simulation are blue rectangles.


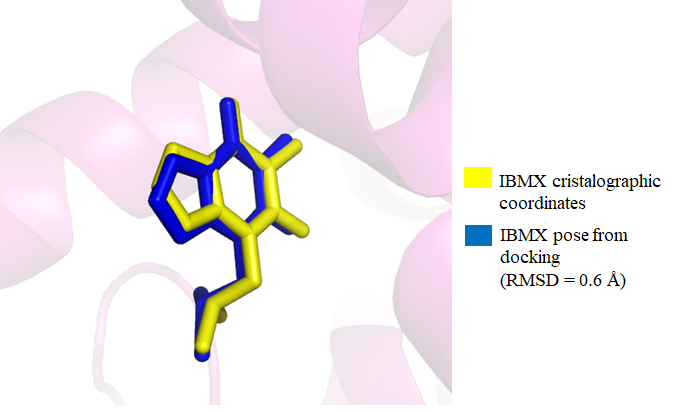


**Figure 2S.** Overlap of the 3-isobutyl-1-methylxanthine (IBMX) coordinates obtained from the crystallographic complex with *Lmj*PDEB1 (PDB 2RQ8) (represented in light yellow stick) with respect to the best scored pose obtained from molecular docking (shown in dark blue stick). RMSD = 0.6 Å. *Lmj*PDEB1 represented in cartoon.

**Table 1S.** Per-residue MMPBSA binding energy decomposition (> ±2 kJ/mol) for the LmjPDEB1-LASSBio1386 complex.

| **Residues**  **Number** | **ΔE_vdw +_ ΔE_elec_** | **± error** |  | **ΔG_pol_** | **± error** | **ΔG*_nonpol_*** | **± error** | **ΔE_binding_** | **± error** |
| --- | --- | --- | --- | --- | --- | --- | --- | --- | --- |
| Asp-796 | 8.58 | 0.09 |  | -0.56 | 0.06 | 0.00 | 0.00 | 8.02 | 0.06 |
| Glu-754 | 3.62 | 0.04 |  | 1.96 | 0.03 | 0.00 | 0.00 | 5.58 | 0.06 |
| Asp-722 | 1.69 | 0.03 |  | 3.28 | 0.05 | 0.00 | 0.00 | 4.97 | 0.06 |
| Asp-835 | 1.08 | 0.03 |  | 3.21 | 0.05 | 0.00 | 0.00 | 4.29 | 0.07 |
| Glu-856 | 1.14 | 0.05 |  | 2.75 | 0.04 | 0.00 | 0.00 | 3.89 | 0.06 |
| Glu-864 | 4.28 | 0.06 |  | -0.48 | 0.03 | 0.00 | 0.00 | 3.79 | 0.04 |
| Lys-897 | 2.42 | 0.08 |  | 0.25 | 0.03 | 0.00 | 0.00 | 2.67 | 0.06 |
| Arg-921 | 2.12 | 0.03 |  | 0.11 | 0.01 | 0.00 | 0.00 | 2.23 | 0.03 |
| Pro-873 | -3.24 | 0.05 |  | 1.28 | 0.02 | -0.15 | 0.00 | -2.11 | 0.04 |
| Lys-832 | -2.83 | 0.04 |  | 0.36 | 0.02 | 0.00 | 0.00 | -2.48 | 0.03 |
| Lys-735 | -3.17 | 0.04 |  | 0.38 | 0.02 | 0.00 | 0.00 | -2.78 | 0.04 |
| Asn-880 | -14.98 | 0.10 |  | 12.49 | 0.08 | -0.51 | 0.01 | -2.98 | 0.09 |
| Ala-798 | -2.89 | 0.07 |  | 0.03 | 0.02 | -0.43 | 0.01 | -3.30 | 0.06 |
| Phe-875 | -5.85 | 0.06 |  | 2.62 | 0.03 | -0.31 | 0.01 | -3.54 | 0.05 |
| Asp-892 | -3.97 | 0.05 |  | -0.16 | 0.03 | 0.00 | 0.00 | -4.13 | 0.04 |
| Gly-889 | -5.56 | 0.05 |  | 1.77 | 0.03 | -0.39 | 0.00 | -4.18 | 0.05 |
| Lys-799 | -5.66 | 0.09 |  | 0.45 | 0.06 | 0.00 | 0.00 | -5.20 | 0.07 |
| Leu-872 | -5.55 | 0.05 |  | 0.07 | 0.02 | -0.53 | 0.01 | -6.02 | 0.05 |
| Phe-857 | -9.12 | 0.07 |  | 2.65 | 0.03 | -0.76 | 0.01 | -7.23 | 0.06 |
| Phe-893 | -10.35 | 0.10 |  | 2.66 | 0.04 | -1.19 | 0.01 | -8.88 | 0.08 |
| Phe-890 | -13.04 | 0.08 |  | 3.29 | 0.05 | -0.69 | 0.01 | -10.45 | 0.07 |
| Met-874 | -18.54 | 0.12 |  | 8.97 | 0.08 | -1.38 | 0.01 | -10.94 | 0.09 |

**Table 2S**. H-bonding interaction over the productive MD time (A) and occurrence of hydrogen bonds between Asn-880 and LASSBio-1386 over time simulation.

| **LmjPDEB1 residues** | **His-800** | **Tyr-858** | **Met-874** | **Asn-880** |
| --- | --- | --- | --- | --- |
| % of MD simulation a H bond to LASSBio1386 is formed | 60.4 | 0.17 | 0.8 | 97.8 |
